# LAMP kit for diagnosis of non-falciparum malaria in *Plasmodium ovale* infected patients

**DOI:** 10.1186/s12936-016-1669-8

**Published:** 2017-01-07

**Authors:** Juan Cuadros, Alexandra Martin Ramírez, Iveth J. González, Xavier C. Ding, Ramon Perez Tanoira, Gerardo Rojo-Marcos, Peña Gómez-Herruz, Jose Miguel Rubio

**Affiliations:** 1 Department of Clinical Microbiology and Parasitology, Hospital Príncipe de Asturias, 28805 Alcalá de Henares, Madrid Spain; 2 Department of Clinical Microbiology and Parasitology, Hospital de la Princesa, Madrid, Spain; 3 Foundation for Innovative New Diagnostics (FIND), Geneva, Switzerland; 4 Department of Medicine and Laboratory, Gambo Rural General Hospital, Kore, West-Arsi, Gambo, Ethiopia; 5 Department of Otorhinolaryngology-Head and Neck Surgery, University of Helsinki and Helsinki University Hospital, Helsinki, Finland; 6 Department of Internal Medicine, Hospital Príncipe de Asturias, Madrid, Spain; 7 Malaria and Emergent Protozoa Laboratory, National Center for Microbiology of Majadahonda, Madrid, Spain

**Keywords:** Malaria, LAMP, Loop-mediated isothermal amplification, *Plasmodium ovale*

## Abstract

**Background:**

Microscopy and rapid diagnosis tests have a limited sensitivity in diagnosis of malaria by *Plasmodium ovale*. The LAMP kit (LoopAMP®) can be used in the field without special equipment and could have an important role in malaria control programmes in endemic areas and for malaria diagnosis in returned travellers. The performance of the Pan primer of the kit in detecting malaria by *P. ovale* was compared with the results of standard nPCR in samples of patients returning from *P. ovale* endemic areas.

**Methods:**

*Plasmodium ovale* positive samples (29, tested by PCR and/or microscopy) and malaria negative specimens (398, tested by microscopy and PCR) were collected in different hospitals of Europe from June 2014 to March 2016 and frozen at −20 °C. Boil and spin method was used to extract DNA from all samples and amplification was performed with LoopAMP® MALARIA kit (Eiken Chemical, Japan) in an automated turbidimeter (Eiken 500). The results of LAMP read by turbidimetry and with the naked eye were compared.

**Results:**

The kit showed a sensitivity of 100% and a specificity of 97.24% with positive and negative predictive values of 72.5 and 100%, respectively. Naked eyed readings were in accordance with turbidimetry readings (sensitivity, 92.5%, specificity, 98.96% and positive and negative predictive values, respectively, 90.24 and 99.22%). The limit of detection of LAMP assay for *P. ovale* was between 0.8 and 2 parasites/µl.

**Conclusions:**

The Pan primer of the Malaria kit LoopAMP® can detect *P. ovale* at very low-levels and showed a predictive negative value of 100%. This tool can be useful in malaria control and elimination programmes and in returned travellers from *P. ovale* endemic areas. Naked eye readings are equivalent to automated turbidimeter readings in specimens obtained with EDTA.

**Electronic supplementary material:**

The online version of this article (doi:10.1186/s12936-016-1669-8) contains supplementary material, which is available to authorized users.

## Background

Malaria incidence has recently been declining globally, although in many African countries it is still one of the top health problems. The efforts to control and eradicate malaria will still need a strong diagnostic capacity, which allows any parasitaemic patient to be detected and treated sooner. LAMP is a simple molecular diagnostic method based on the principle of isothermal amplification, which does not require special equipment or special distribution in laboratories, and provide results in 60 min [1]. Different clinical studies have validated this rapid molecular test in the field with a performance similar to conventional PCR [2, 3]. Additional advantages of LAMP are its tolerance to inhibitory substances present in blood samples (such as haemoglobin and immunoglobulin) [4] and the possibility of being used also on small amounts of blood on filter papers. Furthermore, it could be combined with simple techniques such as microwave DNA extraction [5] in basic laboratories in low resource settings. On the other hand, the growth in international travel and migration has increased the incidence of imported malaria cases in developed countries. In Spain, submicroscopic malaria is common in sub-Saharan migrants (up to 35.5% in one series [6]) and *Plasmodium ovale* infection may represent up to 8% of imported malaria cases, as shown in some published series of mainly West African patients [7]. Therefore, sensitive molecular tools are needed both for malaria control programmes and for detecting with certainty malaria imported cases in patients returning from *P. ovale* endemic areas.

LAMP has showed excellent sensitivity and specificity in diagnosing *Plasmodium falciparum* and *Plasmodium vivax* malaria, but there is little information about the performance of the test for other species, such as *Plasmodium malariae*, *P. ovale* and *Plasmodium knowlesi*. Although no specific *P. ovale* LAMP primers is commercially available, the PAN primer of the LoopAMP® test could be used for initial detection of *P. ovale* infections.

The primary objective of this study was to determine the diagnostic validity of LoopAMP® in comparison to microscopy and conventional multiplex nested PCR (nPCR) in the diagnosis of *P. ovale* infections in archived clinical specimens. Secondary objectives were to compare the naked eye reading of the amplification products with the automated reading by turbidimetry as well as to determine the specificity of the *P. falciparum* LoopAmp® primer in specimens of patients infected with *P. ovale*.

## Methods

The study protocol was previously approved by the Ethics Committee of the Hospital Príncipe de Asturias, Madrid, Spain. Signed informed consents were obtained from all individuals with *P. ovale* malaria from whom blood samples were collected.

### Sample collection

The sample size of positive *P. ovale* samples was limited by the availability of positive specimens (n = 30; *P. ovale wallikeri* 13, *P. ovale curtisi* 8, unknown subspecies 9). These samples were collected with EDTA from venous blood in patients with confirmed *P. ovale* malaria by microscopy and/or PCR and then frozen at −20 °C. No mixed infections were included in this study. The positive specimens were obtained in different European hospitals for routine testing between June 2014 and March 2016 in the context of an ongoing prospective clinical study on *P. ovale* infections which is being developed in Spain and Europe [7]. The number of negative specimens to be tested was established in 411, according to the prevalence of *P. ovale* imported malaria in our hospital (HUPA) in relation with other species (6.8%, 18 out of 263 total malaria cases attended in the period 2007–2015) and the total population studied (1.7% of 1007 patients with suspected malaria in the same period). These negative specimens were obtained from patients coming from West and Central Africa who attended Spanish hospitals with a febrile syndrome and tested negative in thick films, RDTs and PCR multiplex for malaria [8] and were preserved in the clinical specimens Archive of the Microbiology Department of Hospital Príncipe de Asturias and in the Bio Bank of the Malaria laboratory (C.0001392) of the Spanish National Center for Microbiology of Majadahonda.

### Sample processing

The boil and spin method [9] was used for extracting the DNA from frozen whole blood of all patients in a sample preparation area separated from the amplification area. Briefly, an aliquot of 60 μl of whole blood of each patient was transferred to the extraction tube and mixed with 60 μl of extraction buffer (400 mM NaCl, 40 mM Tris pH 6.5, 0.4% SDS) by vortex for 10 s. Extraction tubes containing the samples were placed in a hot-block (Techne DRI-Block DB) at 95 °C for 5 min, and were subsequently centrifuged at 10,000*g* for 3 min (Jouan MR23). Finally, 30 μl of clear supernatant were transferred to a dilution tube with 345 µl of sterile water (DNA samples were then stored at −20 °C for a maximal period of 2 months for testing).

### LAMP reaction and reading

The commercial Pan and *P. falciparum* LoopAMP® kits (Eiken Chemical Co., ref. LMP561 and LMC562, respectively) were tested with extracted DNA of blood samples collected in EDTA and kept frozen at −20 °C. Since EDTA can produce unspecific fluorescence under UV light, the readings of the amplifications products were performed by automated turbidimetry with a turbidimeter (Eiken 500) and with the naked eye [10]. Due to the possibility of DNA degradation with time, the archived *P. ovale* PCR positive specimens testing negative for LoopAMP® were rechecked by conventional PCR to ascertain DNA viability.

The Pan specific primers detect a target mitochondrial DNA sequence common to all the *Plasmodium* species infecting humans. From the dilution tubes, 30 μl of extracted DNA was added in a reaction tube for Pan or *P. falciparum* and then were shaken following the kit instructions for mixing and dissolving. For each batch of 16 reactions, one amplification positive control and one amplification negative control were included alongside (14 samples and 2 controls). The Eiken 500 Turbidimeter was configured with the settings for malaria reaction (amplification at 65 °C for 40 min and enzyme inactivation at 80 °C for 5 min). All the amplifications reactions were also read in a blind manner with the naked eye for the purpose of comparison with the standard turbidimetry.

### Multiplex nested PCR

All samples were tested previously by a validated multiplex nPCR [8] and repeated, as well by duplicate, in case of discordance with the LAMP result. The primer used target the small subunit (SSU) rRNA gene and involves a sequence of two multiplex PCR amplifications. The first reaction amplifies *Plasmodium* ssrDNA from blood samples infected with malaria and includes a positive reaction control, amplification of the Human ssrDNA gene, which indicates whether the reaction is working properly or not. The second reaction enables the identification of the four human malaria species (*P. vivax*, *P. falciparum*, *P. ovale* and *P. malariae*) for the fragment size of the product amplified, mixed infection yield the corresponding fragments for species involved.

### Limit of detection of the LAMP technique

The limit of detection (LoD) of the Pan primer LAMP assay for *P. ovale* was determined using two whole blood specimens with a known parasitaemia of *P. ovale* by microscopy (8111 and 2015 parasites/µl). Briefly, each sample was serially tenfold diluted down to 0.08 and 0.02 parasites/µl, respectively, with blood from a Spanish patient negative for VIH, VHB, and VHC and no story of travelling to endemic malaria areas. DNA was extracted from each dilution by the boil and spin method and then tested by LAMP twice.

### Statistical methods

Sensitivity, specificity, positive and negative predictive values with 95% confidence intervals (CI) were calculated using the nPCR as the reference test by means of EPI Dat (3.1) (2006) Sergas [Software] [11].

## Results

A total of 427 clinical samples (29 *P. ovale* positive and 398 malaria negative) were used to evaluate the performance of the LoopAMP malaria kit for the detection of *P. ovale* parasites in clinical samples. Of the 30 patients with *P. ovale* malaria previously confirmed by nPCR, one sample was excluded due to lack of amplification by LAMP as well as by nPCR when retested. This is possibly due to degradation of DNA over time or an error in initial diagnosis. The other 29 samples were positive with the Pan probe of LAMP, obtaining a sensitivity of 100% (29/29; CI 95%: 98.3–100%). Of the 398 negatives controls remaining after excluding those samples with degraded DNA and abnormal curves (see Additional file 1), 11 samples were positive with LAMP (nPCR was repeated in these samples and was negative) obtaining a specificity of 97.24% (387/398; CI 95%: 95.5–99%) compared with nPCR. Positive and negative predictive values were, respectively, 72.5% (CI 95%: 57.4–87.6%) and 100% (CI 95%: 99.9–100%) (Table 1).

**Table 1:** Comparison of the Pan primer LoopAmp® with nested PCR (n = 427)

	LAMP positive	LAMP negative
PCR positive	29	0
PCR negative	11	387

Sensitivity 100% (29/29; CI 95%: 98.3–100%); Specificity: 97.24% (387/398; CI 95%: 95.5–99%). PPV and NPV, respectively, 72.5% (CI 95%: 57.4–87.6%) and 100% (CI 95%: 99.9–100%)

**Table 2:** Comparison of reaction readings with the naked eye or the turbidimeter Eiken 500

	NE positive	NE negative
Turbidimetry Pos	37	3
Turbidimetry Neg	4	381

Sensitivity, 92.5% [37/40; CI 95%: 83.09–100%]; Specificity, 98.96% [380/384 CI 95%: 98.81–100%]; PPV and NPV, respectively, 90.24% [CI 95%: 79.94–100%] and 99.22% [CI 95%: 98.20–100%]

The comparison with naked eye (NE) readings was possible in 384 LAMP negative specimens (of 387 negative controls only 3 were indeterminate by NE reading) and 40 turbidimetry positive specimens (29 positive controls and 11 false positive results). All in all, turbidimetry and NE were in good agreement, with only 4 false positive and 3 false negative results by NE: Sensitivity, 92.5% [37/40; CI 95%: 83.09–100%]; Specificity, 98.96% [380/384 CI 95%: 98.81–100%]; positive and negative predictive values, respectively, 90.24% [CI 95%: 79.94–100%] and 99.22% [CI 95%: 98.20–100%] (Table 2).

*Plasmodium falciparum* LAMP reactions were found to be negative in 29 out of 29 confirmed *P. ovale* samples. One *P. ovale* sample positive by *P. falciparum* LAMP was excluded as it was found to be also positive for *P. falciparum* by nPCR. In this testing session, 8 specimens produced initially an abnormal curve (see Additional file 2) which was considered negative. In all these specimens LAMP test was repeated and a flat curve negative test was confirmed.

The LoD of the Pan LAMP assay for the detection of *P. ovale* parasites were determined to be at 0.8 parasites/µl in one of the two samples evaluated and 2 parasites/µl in the other one. Duplicate testing was performed with the same results and similar Tt in the replicates (Additional file 3). Then, it can be assumed a LOD of 2 parasites/µl, as it was the lowest value at which all replicates were positive, with a proportion of 2/4 samples detected at lower values (0.8 parasites/µl).

In the study, an episode of contamination during one of the amplification sessions was detected (unexpected occurrence of false positive results in some tubes in the same day, n = 14). No apparent breaking of the protocols nor incidents during pipetting procedures or leaking could be detected. To solve the problem, before the next amplification session, all working surfaces, equipments and reagents were decontaminated with sodium hypochlorite, a new working space for the amplification reaction was established, and the DNA of all false positive samples was re-extracted from archived specimens. The LAMP test was repeated with all these second DNA samples and the number of false positive results was reduced to 5.

## Discussion

Malaria control and eradication programmes worldwide require reliable tools for detecting very low parasite densities in asymptomatic patients, where microscopy and RDTs can produce false negative results, as it is the case in areas of low transmission where submicroscopic malaria can be a key factor in the control programmes [12, 13]. Non *P. falciparum* infections have lower parasite densities than *P. falciparum* infections [12] and the sensitivity of RDTs in *P. ovale* and *P. malariae* infections can be very low [14]. On the other hand, for travellers returning from *P. ovale* endemic countries, an affordable molecular rapid test which no requires special equipment with a very high negative predictive value for all common species of malaria could be very useful.

In this study, it is demonstrated the accurate and sensitive detection of *P. ovale* parasites using *Plasmodium* genus-specific LAMP assay. This is the hitherto largest reported study about LAMP in *P. ovale* malaria.

The obtained results confirm the high sensitivity and specificity of LAMP (100 and 97.2%, respectively) for detecting *P. ovale* infected patients with the PAN primer when comparing to nested PCR, as other studies showed previously with *P. falciparum* (99 and 93%) in Thailand [15], all five human malaria species in Malaysia (100 and 100%) [16], and in a remote clinic in Uganda (89.5 and 95.9%) [1], suggesting that malaria LAMP is a useful molecular tool for detection of low-density malaria infections, including malaria caused by *P. ovale.* Of the 427 clinical samples tested, only 11 resulted in discordant results between LAMP and multiplex nested-PCR. All these discordant results were false positives compared to nPCR results. However, another explanation for these results classified as “false-positive” could be that they are actually true positive of very-low parasitaemia that were undetected by the nPCR reference standard, as has been previously reported [16].

Naked-eye readings showed a very good correspondence with the results obtained by turbidimetry. This result confirm that LAMP can be used in field settings when there is no turbidimeter available, the blood is extracted with EDTA anticoagulant and the UV fluorescence reading method cannot be used [17].

The limit of detection of *P. ovale* malaria was determined between 2 and 0.8 parasites/µl of blood, even more sensitive than the results obtained in previous studies about LoDs for *P. ovale*, which were performed with malachite green-LAMP or with ultraviolet (UV) light as reading methods (3 and 10, respectively) [16, 18].

The occurrence of a contamination episode could have had an impact in the specificity results, if it had remained undetected. Contamination could be due to the semi-automated nature of the test and the high sensitivity of the LAMP technique as has been also documented in previous studies [1, 15]. In our case, LAMP reaction tubes were tightly closed and never opened after amplification and read out was done by turbidimetry and with the naked eye. Most of the samples were frozen in a different clinical centre, from where the study was completed, and then the DNA was extracted and frozen in the laboratory again until LAMP was done. All these manipulations, which are not common in clinical settings, could have increased the chances of contamination.

No false-negative results were detected and this suggests that the Pan malaria LoopAmp® can be used with confidence in malaria eradication or control programmes where *P. ovale* is endemic, although the kit does not include a set of *P. ovale* specific primers and further testing as nested specific PCR would be necessary for final species identification. However, the specific identification of *P. ovale* in the field would not be crucial, as patients could be treated with chloroquine for a non-falciparum malaria and the final identification could be made later in a reference center.

## Conclusion

The Pan primer of the Malaria kit LoopAMP® can detect *P. ovale* at very low-levels and it showed a predictive negative value of 100%. This tool can be useful in malaria control and elimination programmes and in returned travellers from *P. ovale* endemic areas. Naked eye readings are equivalent to automated turbidimeter readings in specimens obtained with EDTA. The relative simplicity of the LAMP procedure and the low infrastructure costs open a range of opportunities by bringing molecular-level parasite detection and capacity of using malaria LAMP in field settings [1, 17].

## Additional files

**Additional file 1.** Raw data of the study comparing turbidimeter and naked eyed readings.

**Additional file 2.** Abnormal curves obtained by turbidimetry.

**Additional file 3.** Results of the LoD study.

## Abbreviations

LAMP: loop-mediated isothermal amplification
nPCR: nested polymerase chain reaction
EDTA: ethylenediamine tetraacetic acid
