# Supplementary material for: LAMP kit for diagnosis of non-falciparum malaria in Plasmodium ovale infected patients
Source: Malar J. 2017 Jan 7;16:20. doi: 10.1186/s12936-016-1669-8 (PMC5219760; doi:10.1186/s12936-016-1669-8)

ANEX I

1. Abnormal curves interpreted as positive by the turbidimeter but considered negative in control negative specimens tested with the PAN probe

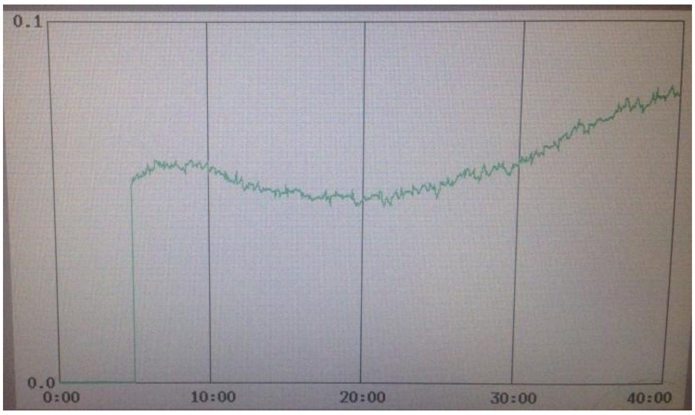

2 . Abnormal curves interpreted as positive by the turbidimeter but considered negative in P ovale positive specimens tested with the Pf probe (black lines)

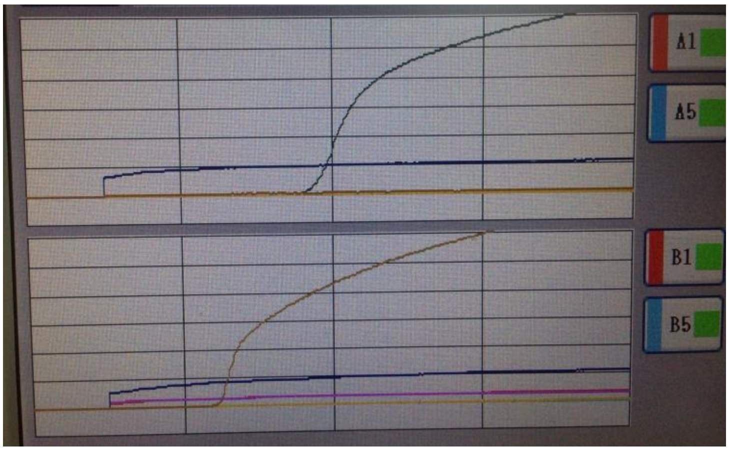

Supplement: Supplementary file 2 — Additional file 2. Abnormal curves obtained by turbidimetry. [file 12936_2016_1669_MOESM2_ESM.pdf]
