# Supplementary material for: LAMP kit for diagnosis of non-falciparum malaria in Plasmodium ovale infected patients
Source: Malar J. 2017 Jan 7;16:20. doi: 10.1186/s12936-016-1669-8 (PMC5219760; doi:10.1186/s12936-016-1669-8)
Supplement: Supplementary file 3 — Additional file 3. Results of the LoD study. [file 12936_2016_1669_MOESM3_ESM.docx]

| Dilution (p/µl) | Tt (Assay 1) | Tt (Assay 2) |
| --- | --- | --- |
| D1 (811,1) | 14.54 | 14.18 |
| D2 (81,11) | 17.36 | 17.63 |
| D3 (8,11) | 22.48 | 23.01 |
| D4 (0,81) | 29.48 | 30.36 |

Comparison of Tt times of the replicates in Control Specimen Number 1 (8.111 parasites/µl)

Supplementary Data III
